# Combined aCGH and Exome Sequencing Analysis Improves Autism Spectrum Disorders Diagnosis: A Case Report

**DOI:** 10.3390/medicina58040522

**Published:** 2022-04-07

**Authors:** Annaluisa Ranieri, Iolanda Veneruso, Ilaria La Monica, Maria Grazia Pascale, Lucio Pastore, Valeria D’Argenio, Barbara Lombardo

**Affiliations:** 1 CEINGE-Biotecnologie Avanzate, Via G. Salvatore 486, 80145 Naples, Italy; ranieria@ceinge.unina.it (A.R.); venerusoi@ceinge.unina.it (I.V.); lamonica@ceinge.unina.it (I.L.M.); pascale@ceinge.unina.it (M.G.P.); pastore@ceinge.unina.it (L.P.); 2 Department of Molecular Medicine and Medical Biotechnologies, Federico II University, Via Sergio Pansini 5, 80131 Naples, Italy; 3 Department of Human Sciences and Quality of Life Promotion, San Raffaele Open University, Via Di Val Cannuta 247, 00166 Rome, Italy

**Keywords:** a-CGH, whole-exome sequencing, autism spectrum disorder, *CNTNAP2*

## Abstract

*Background and Objectives*: The development and standardization of genome-wide technologies able to carry out high-resolution, genomic analyses in a cost- and time-affordable way is increasing our knowledge regarding the molecular bases of complex diseases like autism spectrum disorder (ASD). ASD is a group of heterogeneous diseases with multifactorial origins. Genetic factors seem to be involved, albeit they remain still largely unknown. Here, we report the case of a child with a clinical suspicion of ASD investigated by using such a genomic high-resolution approach. *Materials and Methods*: Both array comparative genomic hybridization (aCGH) and exome sequencing were carried out on the family trio. aCGH was performed using the 4 × 180 K SurePrint G3 Human CGH Microarray, while the Human All Exon V7 targeted SureSelect XT HS panel was used for exome sequencing. *Results*: aCGH identified a paternally inherited duplication of chromosome 7 involving the *CNTNAP2* gene, while 5 potentially clinically-relevant variants were identified by exome sequencing. *Conclusions*: Within the identified genomic alterations, the *CNTNAP2* gene duplication may be related to the patient’s phenotype. Indeed, this gene has already been associated with brain development and cognitive functions, including language. The paternal origin of the alteration cannot exclude an incomplete penetrance. Moreover, other genomic factors may act as phenotype modifiers combined with *CNTNAP2* gene duplication. Thus, the case reported herein strongly reinforces the need to use extensive genomic analyses to shed light on the bases of complex diseases.

## 1. Introduction

Autism spectrum disorder (ASD) is a heterogeneous group of neurodevelopmental disorders characterized by impaired social interaction and communication as well as repetitive and stereotyped behaviors, interests, and activities, according to the Diagnostic and Statistical Manual of Mental Disorders, Fifth Edition (DSM-V) [1,2]. The worldwide prevalence of autism is around 1–2% in the general population, with an average male to female ratio of 4–5:1 [3,4]. The first clinical features are already observable in early childhood and allow for establishing a diagnosis of ASD between 2 and 4 years [5].

The causes of ASD remain largely unknown; however, several studies have shown that genetic and environmental factors play an important role in the etiology of these disorders. Currently, ASD is considered a complex disorder with multifactorial etiology [1]. Approximately 75% of the patients matching the clinical criteria of ASD show isolated ASD [6], while the remaining 25% show complex and syndromic clinical phenotypes. In the latter group, about 10% of patients exhibit specific clinical phenotypes, such as fragile X syndrome, tuberous sclerosis complex, and Angelman or Rett syndrome [2]. The majority of the remaining complex ASD cases represent a heterogeneous group of numerous clinically diverse presentations, many of which still need to be etiologically explained.

Family and twin studies indicate a strong involvement of genetic factors in the etiology of autism. Indeed, the concordance in monozygotic twins is 70–90%, while in dizygotic twins, it reaches 30%, and in siblings 3–19%. Furthermore, several studies show a recurrence rate of the disease in relatives of an affected person of about 5–8%, with a 25–40 times increase in risk compared to the general population. Although ASDs have a hereditary component in approximately 90% of cases, the genetic factors involved in the pathogenesis are still largely unknown [7]. In recent years, the use of Array Comparative Genomic Hybridization (a-CGH) and whole exome/genome sequencing (WES/WGS) has made it possible to identify common and rare copy number variations (CNVs) or single nucleotide variants (SNVs) in genes that code for proteins involved in brain development, which play an important role in the formation and function of neurons and synapses [8,9,10,11,12]. A genetic etiology is recognized in approximately 25–35% of patients with ASD. The wide application of a-CGH and WES allows the increase in diagnosis in patients with ASD and the identification of new candidate disease genes associated with ASD [13,14].

## 2. Case Report

### 2.1. Case Presentation

**Figure 1:**
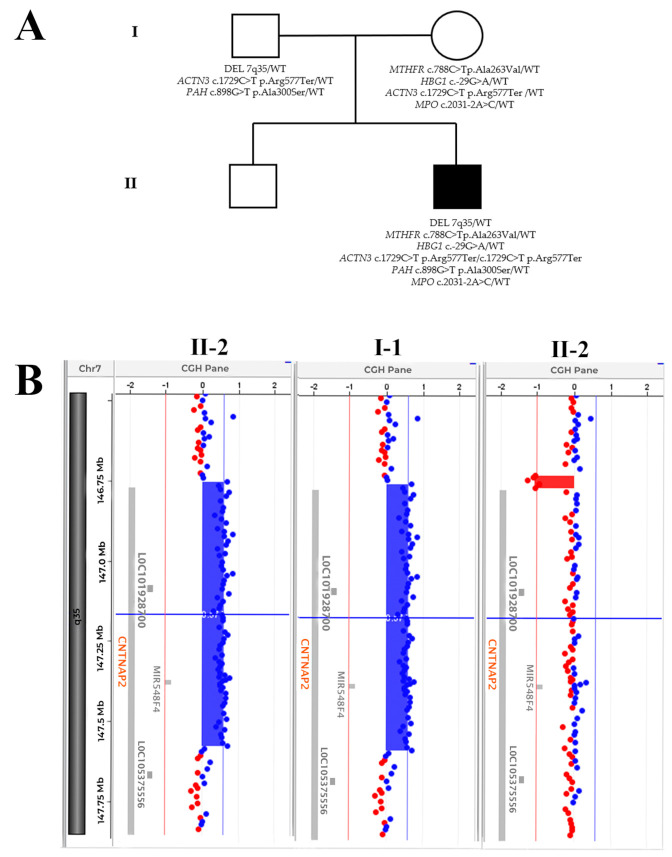
(**A**) Pedigree of the family. Genomic variants identified by both aCGH and WES are reported for each member of the family trio. (**B**) a-CGH profile of chromosome 7. This analysis shows a heterozygous duplication in 7q35 of 883.54 kb involving *CNTNAP2, LOC101928700*, *MIR548F4* in the proband (II-2) and in the father (I-1), CGH analysis. The deletion was not observed in the mother (I-2). The duplication, when present, is indicated by a blue rectangle.

We report the case of a male child of 3 years and 7 months, the second child of healthy unrelated parents (Figure 1A), born at the end of a normal pregnancy, diagnosed with ASD. No family history of neuropsychiatric diseases was reported. The electroencephalogram (EEG) performed revealed values at the normal limits, as well as the Auditory Brainstem Response (ABR) test. The karyotype performed was normal, and the X fragile syndrome test was negative. Nevertheless, at the age of 2, the patient presented significant difficulties in social interaction and communication; indeed, he pronounced his first words around the age of 2 and a half years. At the time of this study, he pronounced about ten words. The functional profile that emerged from the observation of adaptive behavior, assessed by the Vineland scales and spontaneous play, showed fleeting eye contact, not always present. Furthermore, the game is not well-modulated, is unstructured, and is repetitive with participation difficulties and shared game activities. At the age of 3, the autism diagnostic observation schedule—second edition (ADOS 2) was performed and indicated a moderate risk of autism disorder. The Leiter test revealed a normal IQ. The emotional contagion test (TCE) revealed a score of 2. Global motor skills are characterized by the presence of strong psychomotor instability and by the presence of some motor stereotypes that affect exploratory skills. Language is absent; there is the presence of atypical behaviors and a selective diet conditioned by gastroenterological problems. Due to the tendency towards isolation and the lack of interest in other children, the child is currently undergoing neuropsychomotor therapy, mainly aimed at soliciting greater attention to others and the surrounding environment and improving the acquisition of relational skills through the broadening of social interactions and contextual understanding.

### 2.2. Molecular Analyses

A peripheral blood sample was taken from the proband and his parents for molecular analysis using a-CGH and WES. Written informed consent was obtained from all the study participants. DNA samples were extracted from peripheral blood with the Maxwell RSC Blood DNA kit (Promega, Madison, WI, USA). The trio was analyzed using the 4 × 180 K SurePrint G3 Human CGH Microarray (Agilent Technologies, Santa Clara, CA, USA), with an average spacing of 13 kb, allowing an average resolution of 25 kb. The microarray was scanned on an Agilent G2600D scanner. Image files were quantified, and data were visualized using Agilent’s Cytogenomics software (V.4.0.3.12), and the human assembly utilized was GRCh38, hg38 (http://www.ensembl.org/, accessed on 18 March 2022). The CNVs contained in the interval-based report generated by Agilent’s Cytogenomics software were analyzed using Alissa bioinformatic software (Agilent) consulting Clinvar (https://www.ncbi.nlm.nih.gov/clinvar/, accessed on 18 March 2022), Decipher (https://decipher.sanger.ac.uk/, accessed on 18 March 2022), Database of Genomic Variants (http://dgv.tcag.ca/dgv/app/home, accessed on 18 March 2022), GeneCards (http://www.genecards.org/, accessed on 18 March 2022), and OMIM (https://www.omim.org/, accessed on 18 March 2022). This aCGH analysis, carried out on the trio, highlighted the presence of a heterozygous duplication of paternal origin on chromosome 7, in the q35 region, ranging from position 146,754,255 to 147,587,796 (human assembly GRCh38), with an extension of 883.54 kb, involving Contactin Associated Protein 2 (*CNTNAP2,* RefSeq # NC_000007.14), *LOC101928700*, and *MIR548F4* genes (Figure 1B). The duplication includes *CNTNAP2* exons from 2 to 12 and involves parts of introns 2 and 13.

Moreover, WES was carried out on this family trio. A DNA library was prepared for each DNA sample using the Human All Exon V7 targeted SureSelect XT HS enrichment system (Agilent Technologies, Santa Clara, CA, USA), according to the manufacturer’s instructions and as previously reported [8,9]. Next-generation sequencing (NGS) was performed using a Mid Output flow cell v2.5 (300 cycles) on the NextSeq 500 instrument (Illumina, San Diego, CA, USA). Sequence data analysis was carried out using the Alissa bioinformatic software (Agilent) that allows primary and secondary NGS data analysis by integrating 2 sequential pipelines. First, FASTQ data were mapped against the reference human genome sequence using the Align and call tool. The obtained vcf files/sample were then imported into the Interpret module to apply a prioritization tree that allows the identification of the clinically interesting variants for further evaluations. In this case, the “germline trio” option was selected to simultaneously evaluate the results obtained in the family following variants inheritance in the proband. Prioritized variants were also searched on dbSNP (https://www.ncbi.nlm.nih.gov/snp/, accessed on 18 March 2022) and ClinVar (https://www.ncbi.nlm.nih.gov/clinvar/, accessed on 18 March 2022) databases. Their pathogenicity was further assessed using the Varsome tool (https://varsome.com, accessed on 18 March 2022). Through WES analysis, we obtained an average of 1.9 Gb/sample, being equivalent to about 34 million sequences/sample. After reads alignment and variants calling, about 50 k variants/sample were identified. The obtained sequencing specifications are summarized in Supplementary Table S1. A prioritization pipeline specific for trios was applied to highlight potentially interesting variants; in this way, 48 variants were identified in the proband and at least one of the parents for further analyses. After individually checking each variant selected in this shortlist on both dbSNP and ClinVar databases and carrying out a pathogenicity prediction using the Varsome platform, a final set, including 5 potentially interesting variants, was identified (Table 1).

## 3. Discussion

Many genes involved in brain development are related to autism spectrum disorder. The emerging synergism between a-CGH and WES is improving the identification of new variants potentially related to this disorder [15,16,17,18,19,20,21,22].

In this case, the *CNTNAP2* gene, located on chromosome 7, cytoband q35 (146,116,002–148,420,998 (GRCh38/hg38), also called *NRXN4*, is characterized by 24 exons, for a total length of about 2,304,636 Mb, with 24 transcripts and 286 orthologues. This gene encodes a member of the neurexin family, which functions in the vertebrate nervous system as cell adhesion molecules and receptors. In particular, *CNTNAP2* is highly expressed throughout the brain and spinal cord. During human brain development, its expression is highest in the frontal and anterior lobes, striatum, and dorsal thalamus. This expression pattern recapitulates the cortico-striato-thalamic circuitry known to modulate higher-order cognitive functions, including speech and language, reward, and frontal executive functions. In the human cortex, *CNTNAP2* is expressed in layers II–V with enrichment in Broca’s area and other perisylvian brain regions. The enriched expression of *CNTNAP2* in these brain regions, known to be important for speech and language, is consistent with the emerging role of *CNTNAP2* in normal language development in humans [23].

*CNTNAP2* has been implicated in multiple neurodevelopmental disorders, including Gilles de la Tourette syndrome [24], schizophrenia, epilepsy, autism, attention-deficit/hyperactivity disorder (ADHD), and intellectual disability [25]. In particular, several studies show a genetic association of the *CNTNAP2* gene with the autism spectrum disorder [26,27,28]; in fact, the Simons Foundation Autism Research Initiative (SFARI) database (https://gene.sfari.org, accessed on 18 March 2022), which includes all the risk genes for autism, reports it as a strong syndromic candidate. This category includes genes whose variants show a functional effect in genome-wide association studies and whose mutations are related to an increased risk of developing autism. In particular, the *CNTNAP2* gene has a score of 2, which underlines that variants present in this gene could have a functional effect. Studies on mouse models have shown that alterations in candidate genes for ASD (including *CNTNAP2*) induce an altered density of dendritic spines that depends on the specific genetic modification, age, and brain region [29]. A study conducted by Scala et al. reported the presence of a 402 kb duplication of maternal origin in *CNTNAP2* in a child with ID, ADHD, ASD, and speech impairment. These phenotypic traits correlate with *CNTNAP2* deficiency disorder. The CNV of maternal origin involved a complete duplication of exon 2, which may negatively impact protein folding and/or protein-protein interactions. In particular, exon 2 encodes part of the discoidin homology domain, which is found in the extracellular portion of CNTNAP2 and mediates protein-protein interactions [30]. In the proband analyzed in this study, the duplication involves exon 2. It is bigger than that reported in the study conducted by Scala et al., which could support the impact on the patient’s clinical features. Furthermore, other studies reported that rare functional variants, particularly a pathogenetic duplication of 726 kb, in *CNTNAP2*, could significantly increase the risk of comorbid epilepsy/tics in patients with non-syndromic ASD [31]. Finally, the DatabasE of genomiC varIation and Phenotype in Humans using Ensembl Resources (DECIPHER) database (https://decipher.sanger.ac.uk/, accessed on 18 March 2022) reports a 746.21 kb duplication in heterozygosity of likely pathogenic significance (Patient: 424,731) ranging from position 146,754,255 to 147,500,463 with unknown parental origin. Moreover, a second patient (Patient: 255,117) with intellectual disability showed a smaller duplication of 289.15 kb (146,981,175–147,270,327) of maternal origin and uncertain clinical significance, and 3 patients affected by intellectual disability and speech impairment, showed smaller duplications that include the *CNTNAP2* gene (Patients: 385,944, 284,994, 281,827). Only in patient 385,944 was the alteration of paternal origin; the others are of unknown origin. Although the number of *CNTNAP2* gene deletions with clinically relevant significance is higher than duplications, as observed in the literature and by databases such as DECIPHER, the crucial role that duplications may play in the clinical manifestation of neurodevelopmental disorders cannot be excluded. It would be useful to understand if the variants identified in the patient have functional consequences on protein folding or protein/protein interactions. Furthermore, changes in the *CNTNAP2* gene dosage have been shown to be associated with neurodevelopmental disorder-related phenotypes [32]. We detected that the same alterations in the father have no significant clinical symptoms. However, we cannot completely exclude the presence of incomplete penetrance or variable expressivity.

Within the variants identified by WES, only one was in homozygous status, the two parents being both carriers: the c.1729C>T p.(Arg577Ter) (aka R577X) in the exon 15 of the *ACTN3* gene. This variant is a nonsense substitution able to cause a premature stop codon. It is currently reported as a variant of uncertain significance (VUS) both in ClinVar and according to ACMG/AMP (American College of Medical Genetics/Association for Molecular Pathology) classification. *ACTN3* encodes for alpha-actinin-3, a protein expressed in type-II muscle fibers, and that has been associated with performance phenotype, including speed, exercise adaptation, exercise recovery, and sporting injury risk [33]. In particular, the R577X (rs1815739), found in our proband, is considered a common polymorphism due to its frequency, even if it causes an alpha-actinin-3 deficiency [34]. It has been estimated that X allele frequency has been positively selected due to humans’ migration from Africa to Eurasia, thus suggesting that alpha-actinin-3 deficiency may be beneficial [35]. Indeed, even if alpha-actinin-3 deficiency impairs muscles functions and metabolism, it does not result in disease [35]. The XX homozygous genotype is usually poorly represented in power performance athletes. Recently, it has been suggested that this genotype may exert some positive effects on aging, bone metabolism, and also genetic diseases related to muscle impairment, such as McArdle disease and Duchenne dystrophy [35]. Thus, based on current knowledge, this variant does not seem related to the patient’s phenotype but could have a protective significance.

Within the remaining 4 variants, one is a common functional Methylenetetrahydrofolate reductase (*MTHFR*) polymorphism associated with an increased risk of hyperhomocysteinemia that is, in turn, a risk factor for different cardiovascular diseases [36]. This variant may be an important risk factor. Indeed, it encodes a thermolabile enzyme that is less active at high temperature. It has been reported that, in the presence of fever, the hyperhomocisteinemia in the variant’s carriers can impair patients’ cognitive and behavioral skills by modulating NMDAR activity [37,38,39,40]. Moreover, *MTHFR* variant c.788C>T p.(Ala263Val) has been reported as a risk factor for schizophrenia development (Table 1), and it is emerging that several risk genes are shared between schizophrenia and ASDs [41].

The last 3 variants are heterozygous variants classified as pathogenic and found in the *HBG1*, *PAH,* and *MPO* genes. All of them are related to recessive diseases, thus suggesting a carrier status for these diseases.

Finally, since a-CGH analysis has highlighted a duplication in a region of chromosome 7 in the proband that has been inherited from the father and involves the *CNTNAP2* gene, we more thoroughly analyzed this gene to detect the presence of additional variants of maternal inheritance that may contribute to diseases development in the proband. As shown in Table 2, we found 6 variants in this gene, 4 being inherited from the father, 1 from the mother, and the latter being in homozygous status. Both the 2 *CNTNAP2* variants also present in the mother are common polymorphisms currently associated with a benign clinical significance.

## 4. Conclusions

In recent years, great progress has been made in the attempt to clarify the etiology of ASD. The significant percentage of familial cases associated with a high concordance rate in monozygotic twins supports a genetic basis for neurodevelopmental disorders and ASD in particular. On the other hand, the functional significance of CNVs affecting single genes and/or genomic regions is still remarkably incomplete, making diagnostic processes and genetic counseling difficult. Consequently, the discovery of significant genes, which can be considered causal or associated with ASD, represents a necessary step in the diagnostic procedure for a better understanding of their pathophysiology. In fact, a deeper knowledge will help to better classify the clinical characteristics of patients and the mechanisms underlying their clinical manifestations. In particular, a-CGH and WES represent important tools for identifying new disease genes and new alterations in causative or disease-associated genes. This, in turn, will improve the correlations between alterations affecting certain genes and specific phenotypic characteristics, expanding knowledge of the molecular basis of these complex disorders. The interaction between a-CGH and NGS panels or WES in individuals with ASD may be considered an added value to the diagnostic process, although not yet available in routine clinical diagnostic testing. In the case described herein, we detected a *CNTNAP2* duplication by aCGH that may be related to the patient’s phenotype. Since it is paternally inherited, even though we cannot exclude the incomplete penetrance of this variant, we hypothesized the presence of additional genetic variants able to impact clinical phenotype. Thus, we carried out WES and identified some potentially interesting variants. Even if WES analysis did not highlight a pathogenic variant that may explain the clinical phenotype of the patient described herein, we underline again the need to integrate diagnostics to improve the comprehension of the molecular bases of complex diseases. First, the absence of pathogenic variants identified by WES analysis may reinforce the role of the CNV alteration. Moreover, risk variants able to act as phenotype modifiers may also be highlighted as contributing to the final clinical phenotype observed in the patients. Finally, it has to be noticed that variants classification varies over time and may change due to knowledge increase. Thus, a re-evaluation of the genomic data may clarify the molecular bases of the observed disease in the future.

Considering the wide heterogeneity of these disorders, both in terms of behavioral and genetic characteristics, further efforts are needed to explore the possible role of environmental and epigenetic elements. Future studies in close collaboration between divisions of neuropsychiatry and genetics laboratories should be necessarily conducted to shed light on these aspects and their complex connections, thus supporting a better ASD patient diagnosis and management.

## Figures and Tables

**Table 1:** List of the potentially interesting DNA variants identified in the proband after exome sequencing data analysis.

Chr	Gene	cDNA *	Protein *	Reference SNP ID	Status	Inheritance	Associated Phenotype ^†^	Clinvar Classification	ACMG/AMP ^§^ Classification
1	*MTHFR*	c.788C>T	p.Ala263Val	rs1801133	Het	F	Homocystinuria due to MTHFR deficiency—AR Neural tube defects, susceptibility to—AR Schizophrenia, susceptibility to—AD Thromboembolism, susceptibility to—AD Vascular disease, susceptibility to—AD	Drug response	VUS
11	*HBG1*	c.-29G>A		rs368698783	Het	F	Fetal hemoglobin quantitative trait locus 1—AD	Pathogenetic	Benign
11	*ACTN3*	c.1729C>T	p.Arg577Ter	rs1815739	Hom	F + M	Alpha-actinin-3 deficiency—AR Sprinting performance—AR	VUS	VUS
12	*PAH*	c.898G>T	p.Ala300Ser	rs5030853	Het	M	Phenylketonuria AR	Pathogenetic	Pathogenetic
17	*MPO*	c.2031-2A>C		rs35897051	Het	F	Myeloperoxidase deficiency—AR Alzheimer’s disease, susceptibility to—AD	Pathogenetic	Pathogenetic

* According to Human Genome Variation Society (HGVS) guidelines; ^†^ According to MedGen database; ^§^ ACMG, American College of Medical Genetics, and AMP, Association for Molecular Pathology. SNP, single nucleotide polymorphism; ID, identifier; Het, heterozygous; Hom, homozygous; F, father; M, mother; AR, autosomal recessive; AD, autosomal dominant; VUS, variant of unknown significance.

**Table 2:** DNA variants identified in the proband in the *CNTNAP2* gene by exome sequencing.

cDNA *	Protein *	Reference SNP ID	Status	Inheritance	Clinvar Classification	ACMG/AMP ^§^ Classification
c.1897+25A>G		rs2074715	Het	M	Benign	Benign
c.2099-53C>A		rs2538962	Het	F	na	Benign
c.3382-34G>A		rs3801976	Hom	F + M	na	Benign
c.3716-12_3716-9dupCTTT		rs142426153	Hom	F	VUS	VUS
c.3716-6C>G		rs77025884	Het	F	Benign	Benign
c.3723G>A	p.Ala1241=	rs9648691	Het	F	Benign	Benign

* According to Human Genome Variation Society (HGVS) guidelines; ^§^ ACMG, American College of Medical Genetics, and AMP, Association for Molecular Pathology. SNP, single nucleotide polymorphism; ID, identifier; Het, heterozygous; Hom, homozygous; F, father; M, mother; VUS, variant of unknown significance; na, not available.

## Data Availability

The data will be available by contacting the corresponding authors.

## Supplementary Materials

The following supporting information can be downloaded at: https://www.mdpi.com/article/10.3390/medicina58040522/s1, Table S1: Sequencing parameters obtained in each analyzed sample.
