# Supplementary material for: Combined aCGH and Exome Sequencing Analysis Improves Autism Spectrum Disorders Diagnosis: A Case Report
Source: Medicina (Kaunas). 2022 Apr 7;58(4):522. doi: 10.3390/medicina58040522 (PMC9030270; doi:10.3390/medicina58040522)
Supplement: Supplementary file 1 [file medicina-58-00522-s001.zip › medicina-1624190-supplementary.pdf]

Table S1. Sequencing parameters obtained in each analyzed sample.

	P	M	F
Number of sequenced bases	1,923,284,925	1,986,042,438	1,992,418,129
Number of reads	45,485,838	47,220,731	47,622,891
Number of QF on target reads	33,241,855	34,943,225	35,242,003
Percentage of QF on target reads	76,01%	76,97%	76,88%
Median read depth in analyzable target regions	60	68	68
Average read depth in analyzable target regions	66	61	61
Percentage of analyzable target regions covered by at least 20 reads	93,46%	93,05%	93,18%
Total DNA variants	50,146	50,094	49,495
Exonic	26,282	26,507	25,877
Intronic	2,007	1,982	1,946
Filtered Variants		48	

P: proband; M: mother; F: father; QF: quality filters.
